# Occupational Injuries among Latino/a Immigrant Cattle Feedyard Workers in the Central States Region of the United States

**DOI:** 10.3390/ijerph18168821

**Published:** 2021-08-21

**Authors:** Athena K. Ramos, Suraj Adhikari, Aaron M. Yoder, Risto H. Rautiainen

**Affiliations:** 1 Center for Reducing Health Disparities, Department of Health Promotion, University of Nebraska Medical Center, Omaha, NE 68198-4340, USA; 2 Department of Environmental, Agricultural and Occupational Health, University of Nebraska Medical Center, Omaha, NE 68198-4388, USA; suraj.adhikari@unmc.edu (S.A.); aaron.yoder@unmc.edu (A.M.Y.); rrautiainen@unmc.edu (R.H.R.)

**Keywords:** agriculture, feedlot, feedyard, injury, animal handling, Latino/Hispanic, immigrant workers

## Abstract

Agriculture is a dangerous industry with high rates of occupational injuries. Immigrants comprise the majority of the hired agricultural workforce in the United States, and these workers may be at a higher risk for job-related injuries. This study addressed the frequency, characteristics, and risk factors of occupational injuries among Latino immigrant cattle feedyard workers. Data were collected through structured interviews with Latino immigrant cattle feedyard workers in Kansas and Nebraska (*n* = 243; 90.9% male). Descriptive statistics and logistic regression were used to identify risk factors for injury. Nearly three-fourths of participants (71.2%) reported having experienced one or more injuries in the past while working on a cattle feedyard. The most frequent types of reported injuries, including those not requiring medical care, were bruises/contusions (40%), cuts/lacerations (21%), and sprains/strains (12%). These injuries were mainly caused by animals/livestock (33%), chemicals (23%), falls (12%), and tools (9%). Significant risk factors for injury included male gender (OR 5.9), being over age 35 (OR 2.6), working on a large or an extra-large feedyard (OR 5.4), having 11 or more employees on the feedyard (OR 3.6), and working more than eight hours a day (OR 4.7). Having received safety training was also associated with greater risk of injury in a univariable model (OR 2.6). Cattle feedyard workers are at high risk for injury and require more effective preventive measures.

## 1. Introduction

Agriculture is a dangerous industry with high rates of occupational injuries, illnesses, and deaths reported in statistics and studies worldwide [1,2]. Agriculture is often considered a 3-D industry, one that is dangerous, demanding, and dirty. The United States (U.S.) Bureau of Labor Statistics (BLS) annual reports indicate that agriculture has had the highest rates of injuries of any major industry sector in the U.S. over the past two decades. In 2019, the incidence of fatal injuries was 23.1 injuries per 100,000 full-time equivalent (FTE) workers in agriculture compared to 3.5 per 100,000 in all other industries combined [3]. The rate of non-fatal injuries was 5.2 injuries per 100 FTE workers in agriculture and 2.8 per 100 in all other industries combined [4]. Specific subsectors of agriculture, including beef cattle production, had even higher injury rates.

Cattle feedyards are part of the beef production cycle in the U.S. A feedyard is a physical space where beef cattle typically spend between three and six months, and where they are fed a specific ration of grains and other nutrients to help them gain weight, thereby adding muscle and fat [5]. Most cattle feedyards are small operations having less than 1000 head of cattle; however, the large feedyards, representing just 7% of the industry, accounted for 87% of the cattle marketed and sold. Cattle feedyards are concentrated in the Midwestern part of the U.S. Indeed, Texas, Nebraska, and Kansas lead the nation for the number of cattle on feed [6]. More than 53% of all feedyards in the U.S. and almost half of all feedyard workers are located in the Central States region consisting of Iowa, Kansas, Minnesota, Missouri, Nebraska, North Dakota, and South Dakota. In 2018, Nebraska led in the number of cattle feedyard establishments (254) in this region, whereas Kansas had the highest number of cattle feedyard workers (3018) [7].

Depending on the size of the operation, cattle feedyard work may be divided into different departments such as the processing crew, cowboys or pen riders, hospital/sick pen, feedmill, feed delivery, yard maintenance, administration, and security. The processing crew manages the entrance and exit of cattle on and off the feedyard. They may weigh the cattle, provide basic animal health and welfare assessments, and tag the animals. Cowboys or pen riders check cattle health daily by either riding (on horseback or ATV/UTV) or walking through the pens where the animals are housed. If any sick animals are found, they are pulled out of the group and are taken to the hospital pen to be further evaluated. In the hospital/sick pen, animals may receive medications or other special treatments to promote their recovery. At the feedmill, grains and other nutrients are ground and mixed into a specific ration to meet the nutritional needs of the animals. The feed delivery trucks then pick up the feed and drive it around the yard to fill the feed bunks. A yard maintenance team may work in the shop with heavy equipment (e.g., tractors), weld and fix fencing, and handle other maintenance issues as they arise. Administration focuses on the management and business operations of the yard. Finally, the security department ensures the safety of the perimeter of the yard [8].

Cattle feedyards are high-risk work environments for many reasons. First, workers are handling large animals including cattle and depending on the type of work, potentially horses as well. Workers may engage in hazardous activities such as moving cattle in and out of pens, treating cattle in enclosed spaces, and using chutes and gates [9]. Second, workers may slip, trip, or fall doing outdoor work in various risky weather conditions. Animal pens may be wet, muddy, snow-covered, or uneven, and workers could fall from horseback while riding through the pens [10]. Third, workers may drive vehicles (i.e., feed trucks) and use mobile equipment (i.e., skid steers, loaders, ATVs/UTVs). Fourth, workers may be exposed to veterinary pharmaceuticals, pesticides, and other harmful chemical substances, which could have both acute and long-term health effects. Finally, feedyard employers may not have ongoing job-related safety training programs, often solely relying on a safety orientation completed at the time of hire [11].

Many cattle feedyard employers face challenges recruiting the workforce that they need, and cattle industry leaders and safety consultants have noted that immigrants and foreign-born workers are becoming more prevalent in the cattle feeding industry, especially on larger feedyards [12]. It is estimated that approximately 52% of the livestock workforce is foreign-born [13]. This is concerning, as the National Occupational Research Agenda (NORA)’s Agriculture, Forestry, and Fishing Sector Council has deemed immigrant and foreign-born farmworkers as a “vulnerable” worker population due to issues related to English language proficiency, limited job-related training and formal education, and often not having legal authorization to work in the U.S. (i.e., being undocumented) [14,15,16].

The true incidence and severity of injuries to agricultural workers is not well-known, as the injuries are often underreported [17]. Even less is known about the occupational injuries experienced by immigrant workers, as underreporting may be even more prevalent among these workers for fear of losing one’s job, being reported to immigration authorities, or having limited knowledge of worker’s rights and how to navigate the unfamiliar and complex bureaucratic systems in the U.S.

The purpose of this study was to identify the frequency, characteristics, and risk factors for occupational injuries experienced by Latino/a immigrant workers on cattle feedyards.

## 2. Materials and Methods

This article describes injuries reported by participants in the “Health and Safety among Immigrant Cattle Feedyard Workers in the Central States Region” study, conducted between May 2017–February 2020 as part of the Central States Center for Agricultural Safety and Health (CS-CASH) and funded by Centers for Disease Control and Prevention and the National Institute for Occupational Safety and Health (NIOSH).

### 2.1. Participants

To be eligible to participate in this study, individuals had to be at least the age of majority in the state where the data were collected (i.e., Kansas ≥ 18 or Nebraska ≥ 19), identify as a Hispanic/Latino immigrant, and be currently employed on a cattle feedyard in either Kansas or Nebraska. Given the total feedyard employment in the two states of 5990 [7], it was estimated that there were approximately 3115 immigrant workers who would be eligible to participate in the study. A total of 264 workers were actively invited to participate in the study, and 243 workers participated, resulting in a 92% response rate.

### 2.2. Procedures

Latino/a cattle immigrant feedyard workers were recruited to participate in the study through multiple mechanisms including word-of-mouth, flyers posted in community locations, Facebook advertising, and through employers. There were three bilingual and bicultural field research team members who had many years of experience working with immigrant farmworkers who also recruited participants through their social networks in agriculture and ranching as well as through connections with community organizations using a snowball sampling technique. Members of the field research team visited counties with high numbers of feedyard employees to meet with community organizations who assisted in identifying both workers and the locations that workers frequent.

A total of seven members of the field research team (two men and five women) conducted interviews with workers; however, the majority of the interviews (*n* = 223) were conducted by two male members of the team. Team members were trained on techniques to build trust, reduce bias, and ensure the participants’ understanding of the questions. Interviews were scheduled with workers who met the inclusion criteria in an agreed upon location after working hours (i.e., participants’ homes, public library, or restaurant) except for 20 interviews, which were facilitated by employers at the jobsite during working hours. A bilingual and bicultural member of the field research team would travel to conduct each interview face-to-face with the workers, and informed consent was obtained from each participant prior to beginning the research interview. Interviews used a structured questionnaire that explored several health outcomes and contributing factors using questions from validated instruments. It addressed the occupational context; prevention opportunities; physical health; stress, emotional health, and social well-being; and demographics. All study materials were available in English and Spanish, and the workers could participate in the language of their choice. Interviews took approximately 75 min, and a $25 or $30 gift card was given to all participants who completed the interview. Compensation to participants was increased during the study to improve recruitment. The study was approved and considered exempt by the Institutional Review Board at the University of Nebraska Medical Center.

### 2.3. Measures

#### 2.3.1. Injury Outcome and Descriptors

The present analysis is derived from questions within the occupational context, prevention opportunities, and demographic sections of the questionnaire. To identify injury occurrences, respondents were asked, “Have you ever been injured at work on a cattle feedlot?” If participants responded “no”, a follow-up question was asked, “Are you sure you have never cut, bruised, or hurt your back at work? Even if you did not go to a doctor, these are still considered injuries.” If participants responded that they had been injured at work on a cattle feedlot then additional questions were asked to describe the injuries that they had experienced. These questions included the date of the injury, the type of injury, the body part that was injured, the cause of the injury, a description of tasks that the worker was engaged in when the injury occurred, the severity of pain or discomfort from the injury, whether first aid was used or medical care was sought out, lost time related to the injury, and if the injury was reported to their employer. Further, respondents could report if they had a second and third injury, with the same follow-up questions. Information from the three repeated injury question sets were stacked for each respondent. Thus, the injury outcome counted for regression modeling could have a value of 0 (no injuries), 1, 2, or 3 injuries.

#### 2.3.2. Potential Contributing Factors to Injury

The potential predictors for injury were also derived the occupational context, prevention opportunities, and demographic sections of the questionnaire. Injury predictors are described in more detail below.

Sex was recorded as male or female, recognizing that few in this worker population are female. Worker age was recorded as a continuous variable (in years) and dichotomized into over age 35 and 35 or under for the present analyses. English proficiency was assessed, and the four response options were dichotomized into “not at all” and “a little” (limited English proficient) and “somewhat” and “well” (English proficient). The number of hours worked on average per day and the number of days worked on average per week were recorded as continuous variables.

Workers were asked how many cattle were on the feedyard. Based off of their response, the feedyard size was categorized as small (less than 1,000 head of cattle), medium (between 1,000–7,999 head of cattle), large (8,000–32,000 head of cattle), or extra-large (over 32,000 head of cattle) [18]. Participants were asked about the type of work they did at the feedyard (e.g., cowboy/pen rider, processing, mill, feed delivery, equipment and yard maintenance, etc.). They were asked whether they had received work-related training and if so, what type of training had been provided (e.g., in-person training, video, written materials, etc.). They were also asked whether they received any personal protective equipment (PPE) needed for their job from their employer, and if so, what specific types of PPE were provided. Finally, workers were asked about the number of other workers on the feedyard. Although this was recorded as a continuous variable, it was later dichotomized into 11 or more workers or 10 or less workers. This distinction is important because farms that employ 11 or more people may be subject to the enforcement of Occupational Safety and Health Act (OSH Act) standards [19].

#### 2.3.3. Demographic Characteristics

Additional demographic characteristics of participants were recorded, including country of origin, length of time in the U.S. (in years), formal education completion (i.e., elementary, high school, college, etc.), current location (i.e., Kansas or Nebraska), and tenure in agriculture, feedyard work, and with their current employer (in years).

### 2.4. Data Analysis

All data were analyzed using SPSS Version 25 (IBM Corp., Armonk, NY, USA). Descriptive data on injuries were categorized and tabulated. Distributions of potential predictor variables were tabulated for respondents reporting 0, 1, 2, and 3 injuries. Through crosstab statistics in SPSS, odds ratios (OR) and their respective 95% confidence intervals (CI) were calculated. Variables with multiple categories were dichotomized for risk factor analysis. Unadjusted (crude) ORs were calculated to identify risk factors for injury. Predictors that were significant at the *p* < 0.05 level were entered into multivariable analysis, and the final model was constructed using the stepwise (forward) selection process.

## 3. Results

Of the total of 243 feedyard workers who participated in the study, over 60% were working in Nebraska and the remainder were working in Kansas. Most participants (90.9%) were male, and most were from Mexico, followed by Guatemala, and El Salvador.

**Figure 1:**
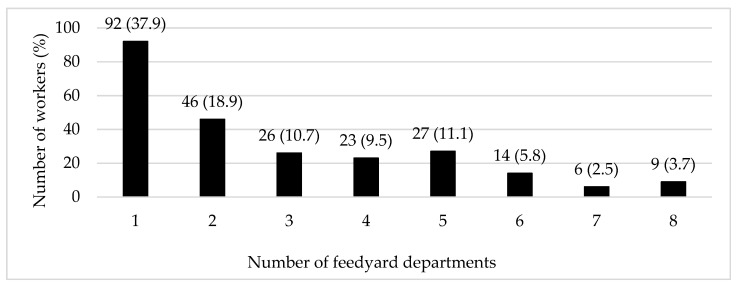
Distribution of feedyard workers by the number of different feedyard departments where they work.

**Figure 2:**
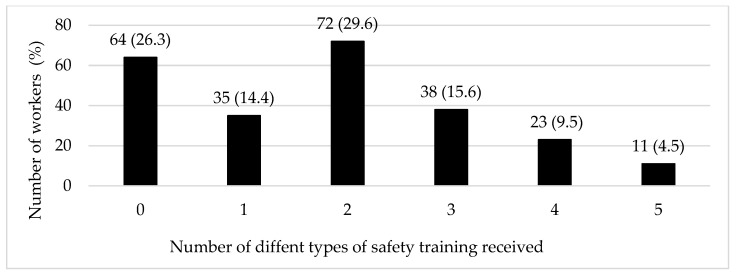
Distribution of feedyard workers by the number of different types of safety training received.

Most participants (62%) performed work tasks associated with two or more feedyard departments such as processing, cowboy/pen riding, yard and equipment maintenance, feed delivery, feed milling, hospital pen, or administration and security. Further, 23.1% of the participants worked tasks in five or more departments (Figure 1). More than one in four workers (26.3%) had not received any type of safety training while 59.6% had received up to three types of safety training (e.g., in-person training, videos, shadowing another worker, hands-on training, and written materials) (Figure 2). Only a few workers (6.2%) had not been provided with any PPE, while nearly one-third (32.9%) had access to five or more types of PPE (e.g., sun protection/sunscreen, sunglasses, gloves, long sleeve shirt, long pants, boots, hearing protection, and face mask).

Table 1 shows the frequency of reported occupational injuries (range: 0 (none) to 3 injuries) among feedyard workers by their demographic characteristics. Overall, the proportion of workers with one or more injuries was high (71.2%). A higher proportion of male workers (75.6%) had injuries compared to female workers (27.3%). Injuries were more frequently reported by workers older than 35 years (80%) compared to younger workers (61.1%). The injury distributions were similar for workers from Mexico and Guatemala, while the numbers of workers from other countries were small. Those speaking English ‘well’ had a lower incidence of injuries (56.5%) compared to less proficient English speakers. Education did not make a clear difference in the distribution of injuries. Over 70% of the participants had been in the U.S. for more than 5 years, but the majority of participants had five years or less of work experience in the cattle feedyard sector (61.8%) and at their current feedyard employer (85.4%). Generally, the proportion of participants ever having an injury in feedyard work increased with the duration of employment in agriculture and feedyard work (Table 1).

Injury characteristics are shown in detail in Table 2. The most frequent types of reported injuries were bruises and contusions (40%), cuts/lacerations (21%), and muscle sprains or strains (12%), which together represented nearly three quarters of the injuries. Injuries were most commonly to the legs/knees/hips (23%), fingers (22%), and hands/wrists (14%). Animals/livestock (33%), tools/equipment (23%), and falls (12%) were the top three causes of injury (Table 2).

Regression analysis of risk factors for injury (Table 3) showed that male workers had much greater adjusted odds (ORa) of being injured (ORa 5.86) compared to female workers. Workers aged 35 years and older had a higher likelihood of injury compared to younger workers (ORa 2.55). Workers on large to extra-large size feedyards had greater odds of being injured (ORa 5.39), compared to small to medium size feedyards. The risk of injury was greater on feedyards with 11 or more employees (ORa 3.56), compared to operations with fewer workers. The risk of injury was greater among participants who worked more than five days a week (vs. fewer days) (ORc 4.58) and among those working eight hours or more per day (vs. fewer hours) (ORa 4.73). Safety training had an adverse association with injury in crude analysis (ORc 2.64). When predictors that were significant in crude analyses at the *p* < 0.05 level were entered into multivariable analysis, the covariates including sex, age, feedyard size, employee count, and work hours per day were significant at the *p* < 0.05 level. English proficiency, number of different jobs performed on the feedyard, and provision of PPE had no significant association with injury.

## 4. Discussion

This study explored the frequency, characteristics, and risk factors of injuries among Latino/a immigrant cattle feedyard workers in the Central States region of the United States. We found that a high percentage of workers (71.2%) reported being injured while working at a feedyard. Few studies have reported injury rates for immigrant workers in agriculture. One study reported 7.9–11.7 injuries per 100 full-time equivalent (FTE) workers among New York and Maine migrant and seasonal farmworkers [20]. In our study, feedyard work experience varied and ranged from less than a year to more than 25 years, and we could not construct similar FTE-based injury rate estimates retrospectively. In a retrospective analysis of insurance records, 52.9% of Finnish farmers had been injured during their work time, which ranged up to 26 years [21]. Reported injury rates among self-employed farmers have varied greatly, ranging from 0.5–42 injuries per 100 farmers a year [22,23]. Recent national statistics on work-related injuries highlight that the beef cattle industry (including feedyards) has nearly double the number of injuries compared to the “all industries” rate (5.3 injuries/100 FTE vs. 2.8/100 FTE in 2019) [4], and this study clearly demonstrates that there are a significant number of injuries in this line of work. Similar to earlier studies of agricultural injuries, the most frequently reported injuries in our study were bruises, cuts, and muscle strains and sprains. The main causes of these injuries included livestock handling, chemicals, falls, and tools.

Previous studies have identified reasons why immigrant workers are vulnerable and at increased risk of illness and injury at work. These concerns include limited host country language proficiency, low literacy, limited formal education completion and job-related training, immigration legal status, risk perceptions, stress, economic challenges and a need to maximize work hours and earnings, limited knowledge of work-related rights, difficult manual labor, working with animals, precarious living conditions, limited access to medical care, and lack of enforcement of safety regulations in agriculture [24,25,26,27,28]. Significant risk factors for injury in our study included male gender, being over age 35, working on a large or extra-large feedyard, having 11 or more employees on the feedyard, and working more than eight hours a day. A systematic review of risk factors for agricultural injury also found male gender, a higher number of workers, and a larger farm size were among risk factors for injury while the association of injury with age and safety training were inconclusive from the results of several studies [29,30]. The association of work time, in hours per day or days per week, has not been well established in agriculture, likely due to the complexities of enumerating working hours for self-employed farmers. However, a recent study of agricultural machine operators in Italy found that work hours were positively associated with unsafe behaviors, leading to near misses, and ultimately, safety incidents [31]. Feedyard employers should consider hiring sufficient workers to be able to meet operational needs so that working beyond a standard shift is not a necessity or a consistent expectation.

Stronger oversight into daily operating procedures on the feedyard is needed. Feedyard employers could ensure greater enforcement of safety protocols and promote safety coaching between supervisors and workers. Regulatory bodies such as the Occupational Safety and Health Administration (OSHA) have a responsibility to enforce and promote worker safety. OSHA has tools, such as the local emphasis program, which is intended to address industries and hazards that pose risks to workers’ safety and health. It uses outreach to industry leaders and employers as well as unannounced inspections to promote and enforce compliance with safety regulations [32]. This program has been used with the dairy industry and focused on the “dairy dozen” (i.e., 12 common dairy safety concerns) [33]. If injury rates in the cattle feedyard sector do not decrease, it is possible that OSHA could establish a local emphasis program focused on feedyards that do not meet the small farm exemption.

Although this study did not address the history of prior injury or sleep deprivation, these may be additional risk factors for occupational injuries. Numerous studies and systematic reviews have reported on the negative effects of fatigue, shift work, and sleep deprivation [34,35,36]. Daily sleep and weekly working hours have been established as risk factors for injury in many studies, including the U.S. National Health Interview Survey [37], and recently, NIOSH established the Center for Work and Fatigue Research to expand the understanding of sources of fatigue such as physically and mentally demanding work, co-morbidities, and environmental concerns [38]. Future research should explore the impact of fatigue and non-standard work on reported agricultural injuries and near misses.

Counter-intuitively, we found that having received safety training was associated with more reports of injuries. However, earlier studies have shown conflicting results as well [29,39]. Safety training focused on increasing knowledge may not be sufficient to bring about changes to behaviors [40]. A systematic review of interventions to prevent injuries in agriculture found no significant effect from educational interventions [41]. The authors speculated that educational interventions such as training may not be strong enough to bring about change unless combined with other behavioral incentives such as financial benefits or legislative requirements. Further, educational intervention trials may suffer from biases favoring control due to non-blinded designs, leaking of educational intervention, and better awareness and willingness to report outcomes among intervention subjects. In our study, it is possible that having received training sensitized workers to be able to recognize and report injuries. Such recognition and reporting may be beneficial to the feedyard industry, as it could lead to a greater understanding of the common conditions and risks experienced by workers, which may result in stronger, more relevant preventive actions over the long term. In our study, this counterintuitive finding of safety training being associated with more injuries may also have been the result of a reactionary approach to safety, including training, where safety measures were implemented after injury incidents had already occurred. Regardless, occupational safety and health training should be provided to all workers at the time of hire and at regular intervals during employment [42]. Training should incorporate best practices for fostering adult learning through engaging, participatory, culturally, and linguistically relevant approaches [43,44,45,46]. Training may also have an added spillover benefit of reducing occupational stress, another potential risk factor for injury [47].

Immigrant farmworkers are a growing worker segment, particularly in animal agriculture, but there is limited data on this population. Published government statistics from the Bureau of Labor Statistics on occupational injuries are likely undercounts [17,48,49]. A recent study conducted by RAND concluded that developing definitions of populations at risk for occupational exposure and profiling them by agricultural commodity, demographic factors, work organization patterns, and worksite tasks should be prioritized [50]. Additional studies are needed to more fully understand occupational risks, injuries, and potential preventive measures across the agricultural industry, but especially among immigrant farmworkers engaged in livestock production.

### Strengths and Limitations

This is one of the few studies that has explored injuries among immigrant livestock production workers in the U.S.; however, there are some limitations to note, including the cross-sectional design, the reliance on self-reports of injury over a long period of time, the selection of potential risk factors, and the limited sample of only Latino immigrant workers in one part of the United States. This study represents a snapshot in time, and causality cannot be determined. Workers may have experienced recall decay, whereby injuries or training sessions further in the past were more likely to be forgotten or underreported; however, our data were based on a series of questions, thereby limiting the possibility of bias. Although our interviewers probed for job-related injuries, it is possible that workers underreported injuries that they had experienced due to self-presentation bias as well. Latino immigrant workers may face differential working conditions compared to other workers based on language and cultural differences as well as immigration legal status, thereby positioning them to work in more dangerous conditions or to complete more risky tasks. Additional studies are needed to understand the influence of these factors on job-related injuries in the cattle feeding industry. Moreover, it is possible that the experiences of Latino immigrant workers in other parts of the U.S., those working in other commodities, and non-Latino workers may differ. Future studies should include objective measures of injury such as those from workers’ compensation claims and OSHA 300 logs [51], conduct onsite observations or use ethnographic approaches to understand injuries and near misses, explore injuries among the greater feedyard workforce, and use longitudinal designs to better understand the influence of training on injury rates and reporting.

## 5. Conclusions

Cattle feedyard work can be dangerous, and the proportion of immigrant feedyard workers reporting injuries at work was exceptionally high compared to previous studies and official published injury statistics. Significant risk factors for injury included male gender, being over age 35, working on a large or extra-large feedyard, having 11 or more employees on the feedyard, and working more than eight hours a day. Because feedyard workers are at a high risk of injury, more effective preventive measures including providing engaging, participatory, culturally, and linguistically appropriate training, greater enforcement of safety protocols, and safety coaching implemented as part of a broader approach to enhance safety culture on feedyards, are needed in addition to ensuring appropriate staffing to meet operational needs.

## Figures and Tables

**Table 1:** Distribution of participants by the number of reported injuries and demographic characteristics.

Characteristics	Count of Reported Injuries *n* (% ^1^)				Total *n* (% ^2^)
	None	1	2	3	
Sex					
Male	54 (24.4)	63 (28.5)	46 (20.8)	58 (26.2)	221 (90.9)
Female	16 (72.7)	2 (9.1)	2 (9.1)	2 (9.1)	22 (9.1)
Age					
35 years or younger	44 (38.9)	28 (24.8)	20 (17.7)	21 (18.6)	113 (46.5)
Older than 35 years	26 (20.0)	37 (28.5)	28 (21.5)	39 (30.0)	130 (53.5)
Country of origin					
Mexico	43 (25.4)	42 (24.9)	40 (23.7)	44 (26.0)	169 (69.5)
Guatemala	9 (21.4)	14 (33.3)	7 (16.7)	12 (28.6)	42 (17.3)
El Salvador	7 (46.7)	5 (33.3)	0 (0.0)	3 (20.0)	15 (6.2)
Other	11 (64.7)	4 (23.5)	1 (5.9)	1 (5.9)	17 (7.0)
English proficiency					
Not at all	15 (31.3)	17 (35.4)	6 (12.5)	10 (20.8)	48 (19.8)
A little	31 (26.3)	31 (26.3)	28 (23.7)	28 (23.7)	118 (48.6)
Somewhat	14 (25.9)	14 (25.9)	10 (18.5)	16 (29.6)	54 (22.2)
Well	10 (43.5)	3 (13.0)	4 (17.4)	6 (26.1)	23 (9.5)
Education					
Never attended	0 (0.0)	0 (0.0)	2 (50.0)	2 (50.0)	4 (1.7)
Grades 1 through 8	26 (32.9)	30 (38.0)	12 (15.2)	11 (13.9)	79 (32.8)
Grades 9 through 11	15 (24.2)	15 (24.2)	15 (24.2)	17 (27.4)	62 (25.7)
High school graduate/GED ^3^	7 (18.9)	5 (13.5)	7 (18.9)	18 (48.6)	37 (15.4)
College 1 to 3 years	4 (44.4)	1 (11.1)	2 (22.2)	2 (22.2)	9 (3.7)
College 4 years or more	17 (34.0)	14 (28.0)	9 (18.0)	10 (20.0)	50 (20.7)
Location					
Kansas	8 (8.4)	19 (20.0)	25 (26.3)	43 (45.3)	95 (39.1)
Nebraska	62 (41.9)	46 (31.1)	23 (15.5)	17 (11.5)	148 (60.9)
Years in the U.S.					
5 years or less	31 (37.3)	25 (30.1)	13 (15.7)	14 (16.9)	83 (34.3)
6–15 years	18 (22.2)	20 (24.7)	22 (27.2)	21 (25.9)	81 (33.5)
16–25 years	11 (20.8)	14 (26.4)	8 (15.1)	20 (37.7)	53 (21.9)
More than 25 years	9 (36.0)	6 (24.0)	5 (20.0)	5 (20.0)	25 (10.3)
Years in agriculture					
5 years or less	32 (43.8)	18 (24.7)	11 (15.1)	12 (16.4)	73 (30.4)
6–15 years	18 (20.0)	25 (27.8)	21 (23.3)	26 (28.9)	90 (37.5)
16–25 years	13 (25.5)	16 (31.4)	8 (15.7)	14 (27.5)	51 (21.3)
More than 25 years	6 (23.1)	5 (19.2)	7 (26.9)	8 (30.8)	26 (10.8)
Years in feedyard work					
5 years or less	55 (36.9)	43 (28.9)	27 (18.1)	24 (16.1)	149 (61.8)
6–15 years	13 (17.6)	17 (23.0)	17 (23.0)	27 (36.5)	74 (30.7)
16–25 years	2 (13.3)	5 (33.3)	2 (13.3)	6 (40.0)	15 (6.2)
More than 25 years	0 (0.0)	0 (0.0)	2 (66.7)	1 (33.3)	3 (1.2)
Years at current feedyard					
5 years or less	62 (30.4)	52 (25.5)	41 (20.1)	49 (24.0)	204 (85.4)
6–15 years	6 (23.1)	11 (42.3)	4 (15.4)	5 (19.2)	26 (10.9)
16–25 years	1 (12.5)	2 (25.0)	1 (12.5)	4 (50.0)	8 (3.3)
More than 25 years	0 (0.0)	0 (0.0)	1 (100.0)	0 (0.0)	1 (0.4)

^1^ Row percentage. ^2^ Column percentage. ^3^ GED is the abbreviation for the General Educational Development test or an equivalent to a U.S. high school diploma.

**Table 2:** Frequencies of reported injuries by injury characteristics.

Injury Characteristics	*n* (%)
Type of injury	
Bruise/contusion	137 (40.3)
Cut/laceration	70 (20.6)
Muscle sprain/strain	39 (11.5)
Injection (accidental stab)	28 (8.2)
Broken bone	25 (7.4)
Burn	10 (2.9)
Inhalation	3 (0.9)
Electrocution	1 (0.3)
Poisoning	1 (0.3)
Other	26 (7.6)
Body part injured	
Leg, knee, or hip	79 (23.4)
Finger	75 (22.2)
Hand/wrist	47 (13.9)
Arm/shoulder	43 (12.7)
Foot	21 (6.2)
Back	17 (5.0)
Head/neck	12 (3.6)
Chest/trunk	11 (3.3)
Eye	11 (3.3)
Toe	3 (0.9)
Other	19 (5.6)
Cause of injury	
Animal/livestock	111 (32.8)
Chemicals	78 (23.1)
Fall	40 (11.8)
Tool or equipment	30 (8.9)
Slip or trip	25 (7.4)
Another worker	12 (3.6)
Chute, gate, or other feedyard structure	2 (0.6)
Vehicle	1 (0.3)
Other	39 (11.5)

**Table 3:** Regression analysis of risk factors for injury.

Characteristics	Not Injured	Injured	Total	ORc (95% CI)	ORa (95% CI)
	*n* (%)	*n* (%)	*n*		
Sex					
Male	54 (24.4)	167 (75.6)	221	8.25 (3.07, 22.13)	5.86 (1.68, 20.36)
Female	16 (72.7)	6 (27.3)	22	Reference	Reference
Age					
Older than 35 years	26 (20.0)	104 (80.0)	130	2.55 (1.44, 4.52)	2.55 (1.22, 5.34)
35 years or younger	44 (38.9)	69 (61.1)	113	Reference	Reference
Feedyard size					
Large/extra-large	50 (23.5)	163 (76.5)	213	7.33 (3.01, 17.88)	5.39 (1.80,16.13)
Small/medium	18 (69.2)	8 (30.8)	26	Reference	Reference
Employee count					
11 or more	41 (21.0)	154 (79.0)	195	5.73 (2.92, 11.24)	3.56 (1.48, 8.59)
10 or fewer	29 (60.4)	19 (39.6)	48	Reference	Reference
Work hours/day					
More than 8 h	40 (21.2)	149 (78.8)	189	4.66 (2.45, 8.83)	4.73 (2.15, 10.39)
8 h or less	17 (68.0)	8 (32.0)	25	Reference	Reference
Work days/week					
More than 5 days	51 (24.2)	160 (75.8)	211	4.58 (2.12, 9.93)	-
5 days or less	19 (59.4)	13 (40.6)	32	Reference	
Safety training					
Received training	42 (23.5)	137 (76.5)	179	2.64 (1.42, 4.90)	-
No training	28 (43.8)	36 (56.2)	64	Reference	
English proficiency					
Somewhat or well	55 (28.2)	140 (71.8)	195	0.85 (0.47, 1.53)	-
Not at all or a little	15 (31.2)	33 (68.8)	48	Reference	
Feedyard jobs					
Multiple departments	49 (32.5)	102 (67.5)	151	0.62 (0.34, 1.12)	-
Single department	21 (22.8)	71 (77.2)	92	Reference	
Provision of PPE					
Provided any PPE	67 (29.4)	161 (70.6)	228	0.60 (0.16, 2.20)	-
Not provided any PPE	3 (20.0)	12 (80.0)	15	Reference	

The “-“ denotes that there was no significant effect at *p* < 0.05 level. ORc: Crude Odds Ratio. ORa: Adjusted Odds Ratio.

## Data Availability

The data presented in this study are available on request from the corresponding author.
